# Simple isocratic method for simultaneous determination of caffeine and catechins in tea products by HPLC

**DOI:** 10.1186/s40064-016-2672-9

**Published:** 2016-07-04

**Authors:** Chamira Dilanka Fernando, Preethi Soysa

**Affiliations:** Department of Biochemistry and Molecular Biology, Faculty of Medicine, University of Colombo, Kynsey Road, Colombo 08, Sri Lanka; College of Chemical Sciences, Institute of Chemistry Ceylon, Adamantane House, 341/22, Kotte Road, Welikada, Rajagiriya, Sri Lanka

**Keywords:** Caffeine, Catechins, HPLC, Isocratic method, Tea

## Abstract

Tea is a popular beverage almost all over the world. Many studies show that tea consumption is closely associated with positive health impact. Most of the HPLC methods used for the determination of tea constituents include gradient elution systems which involve expensive instrumentation. The objective of this study was to develop a simple, rapid precise and low cost HPLC method for the separation and quantification of catechins and caffeine in tea (*Camellia sinensis*). The method utilizes a phenyl column (2.1 × 150 mm) with a UV-detector (280 nm) where excellent chromatographic separation of tea components i.e. gallic acid (GA), caffeine (Caf), epicatechin (EC) and (−)-epigallocatechin gallate (EGCG) was achieved. The isocratic elution system of acetonitrile, glacial acetic acid and deionized water (8:1:91 v/v/v) at a flow rate of 0.5 mL/min was involved. This method produced excellent accuracy and precision. Within run and between run precision was less than 7.5 %. The equations for calibration curves were y = 0.117 (±0.010)x + 0.173 (±0.024), y = 0.100 (±0.003)x + 0.045 (±0.019), y = 0.016 (±0.001)x + 0.006 (±0.004), y = 0.025 (±0.001)x−0.025 (±0.007) for GA, Caf, EC and EGCG respectively. The method validation parameters prove that the method is efficient, a simple and adequate for the quantitative determination of principal components in tea samples.

## Background

Tea is a healthy beverage enjoyed by most population in the world. The medicinal effects of tea include anticancer activity, anti-obesity, antidepressant, anti-inflammatory, antimicrobial etc. (Fernando and Soysa 2015). Tea is a complex mixture of phytochemicals. Among them polyphenols, catechins and caffeine have drawn more attention to study its health benefits. Catechins consist of flavon-3-ol structure and have been proved to be strong antioxidants and free radicals scavenging agents (He et al. 2010). The biological activities of catechins vary depending on their structures. Polyphenolic substances like gallic acid have demonstrated cytotoxic activity in cancer cell lines (Fernando and Soysa 2015). Caffeine, the coexisting component with polyphenols has been found to increase fatty acid oxidation and act as an ergogenic substance (Pesta et al. 2013).

Many HPLC methods have been developed so far to separate and determine catechins and caffeine present in tea and most of them involve with gradient systems (He et al. 2010; Saito et al. 2006). Gradient elution HPLC is complex compared to isocratic system. Some of the major disadvantages of gradient elution systems include the requirement of expensive instrumentation, when getting back to the original concentration, difficulty in optimization of the conditions and obtaining reproducible results (Janoušková et al. 2001; Schellinger et al. 2005; Churácek 1985).

The current method involves simple isocratic method which can be performed in an ordinary laboratory with low cost HPLC machine to determine gallic acid (GA), caffeine (Caf), epicatechin (EC) and (−)-epigallocatechin gallate (EGCG) contents in tea products. This method can be used to analyze the quality of tea and also any adulteration of tea available in the market.

## Results and discussion

**Fig. 1:**
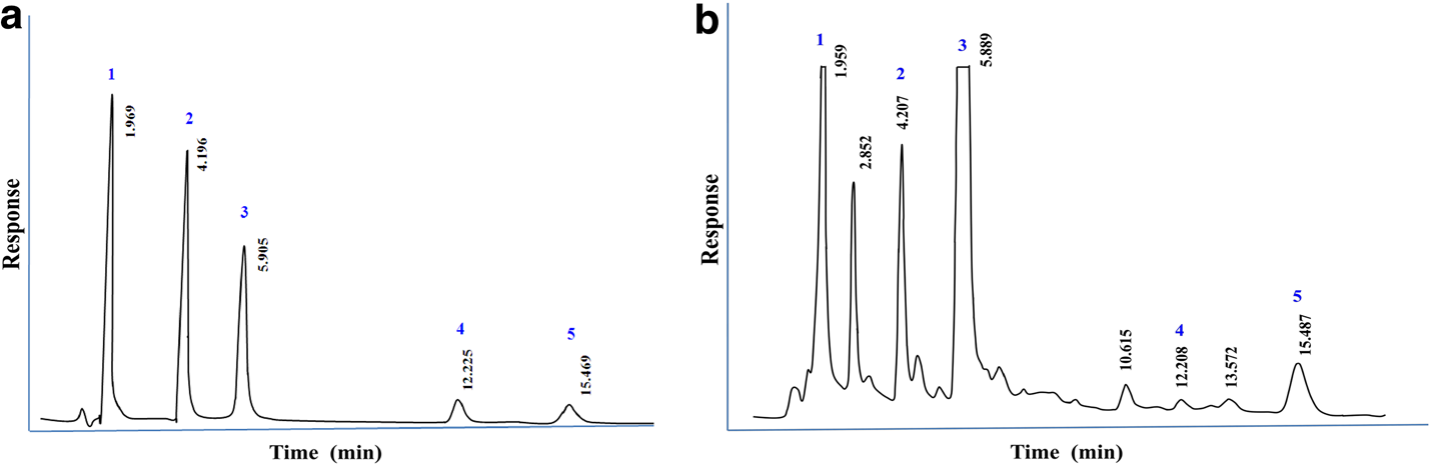
The HPLC chromatograms of standard samples (*1* gallic acid, *3* caffeine, *4* epicatechin, *5* epigallocatechin gallate) each at a concentration level of 8 μg/ml, *2* internal standard i.e., β-hydroxyethyl-theophylline (10 μg/ml) (**a**) and CTC black tea (*1* gallic acid, *2* β-hydroxyethyl-theophylline, *3* caffeine, *4* epicatechin, and *5* epigallocatechin gallate (**b**)

**Fig. 2:**
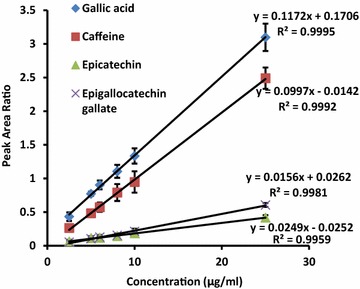
Standard curves obtained for gallic acid, caffeine, epicatechin and epigallocatechin gallate over the concentration range of 2.5–25 μg/ml. (Peak area ratio = Area under curve of the analyte/Area under curve of internal standard)

We developed a simple, rapid and accurate analytical method for quantification of GA, Caf, EC and EGCG in tea products using a low-cost HPLC method. The mobile phase composition and flow rate were optimized to have well defined separation of gallic acid, caffeine, epicatechin, epigallocatechin gallate as well as the internal standard β-hydroxyethyltheophilline with excellent chromatographic resolution (Fig. 1a, b). These compounds were eluted at 1.96, 5.89, 12.21, 15.49 and 4.21 min respectively. The calibration curves were linear over 2.5–25 μg/mL with R^2^ exceeding 0.995 for each of the compounds separately (Fig. 2). The equations for calibration curves were y = 0.117 (±0.010)x + 0.173 (±0.024), y = 0.100 (±0.003)x + 0.045 (±0.019), y = 0.016 (±0.001)x + 0.006 (±0.004), y = 0.025 (±0.001)x−0.025 (±0.007) for GA, Caf, EC and EGCG respectively. The standard deviations of the slope and intercepts of these curves prove a good reproducibility for all substances tested. It also suggests that the concentration used for β-hydroxyethyltheophylline as the internal standard for all the substances i.e. GA, Caf, EC and EGCG analysis is adequate. The accuracy and precision of the present method were sufficient. The accuracy was 96–103 % and the within run and between run precision (CV %) was ranging from 0.8–7.4 % for all the compounds studied. The LOD found was 0.4, 0.7, 1.3 and 0.7 µg/mL, whereas, the LOQ was 1.2, 2.2, 3.8 and 2.2 µg/mL for GA, Caf, EC and EGCG respectively. The slope of the calibration curves remained stable for at least up to 6 weeks suggesting their long term stability when stored at −20 °C with minimal within run and between run variability over the same time period. The summary of validated parameters for the HPLC method used in this study is tabulated in Table 1. In addition to HPLC–UV system, other HPLC based separation systems involved to characterize tea constituents are HPLC-ECD, HPLC-FD, CL-HPLC, HPLC–MS, Ultra HPLC, Nano HPLC and two dimensional UHPLC. Some of these methods show high sensitivity and may be important in analysis of plasma levels of tea catechins. However these systems are expensive, sophisticated and hence requires technical expertise in handling such instrumentation (Yashin et al. 2015). In comparison, isocratic HPLC–UV method developed in the present study is economical and less complicated. The modifications, optimizations and validation we have incorporated in the current method can be applied to analyze GA, Caf, EC and EGCG in tea in an ordinary laboratory.

**Table 1:** Results obtained for the validation of the HPLC/UV method used in the current study

Tea component	Concentration (µg/mL)	Accuracy (µg/mL)	Precision		LOD (μg/mL)	LOQ (μg/mL)
			Within run	Between run		
Gallic acid	3	2.9 ± 0.1	3.8	4.1	0.4	1.2
	4	4.1 ± 0.3	5.1	7.4		
	7	7.2 ± 0.5	3.0	6.6		
	15	15.0 ± 1.0	1.9	7.0		
Caffeine	3	3.1 ± 0.1	2.1	4.6	0.7	2.2
	4	4.0 ± 0.2	3.4	6.4		
	7	7.2 ± 0.4	0.9	5.5		
	15	15.5 ± 0.9	1.6	6.0		
Epicatechin	3	3.0 ± 0.1	2.3	2.0	1.3	3.8
	4	4.3 ± 0.3	3.3	6.9		
	7	6.8 ± 0.4	2.8	5.5		
	15	14.5 ± 1.0	0.8	6.6		
Epigallocatechin gallate	3	3.0 ± 0.1	3.6	3.9	0.7	2.2
	4	3.8 ± 0.1	6.2	2.3		
	7	6.5 ± 0.4	2.7	6.7		
	15	15.4 ± 0.9	2.8	5.9		

## Conclusion

The HPLC method developed is a simple, reliable and rapid to measure GA, Caf, EC and EGCG in tea products. Over 300 sample injections were carried out using a single column without any significant alterations to the peak shape or pressure buildup in the HPLC system.

## Methods

### Reagents and chemicals

Deionized water was used for preparation of all standards and samples. HPLC grade acetonitrile was purchased from BDH (BDH Chemicals Ltd. Poole, England). β-hydroxyethyltheophylline, Caf, GA, EC and EGCG were ACS reagents purchased from Sigma Chemicals, USA. Crush, tear, curl (CTC) low grown pure Ceylon black tea was obtained from Danduwangalawatta Tea factory, Millawitiya, Kuruwita, Sri Lanka.

### Equipment

HPLC was performed with Shimadzu LC 10AS solvent delivery system equipped with UV/VIS variable wavelength detector Shimadzu SPD 10A (Shimadzu Corporation, Japan) and an integrator Shimadzu C-R8A (Shimadzu Corporation, Japan). Chromatographic resolution of components in tea was achieved on a 2.1 × 150 mm, betasil phenyl HPLC column (Thermo scientific). The temperature of the chromatographic column was maintained at 25 °C throughout all experiments. Samples were injected with a syringe loading injector fitted with a 100 μL loop. Shimadzu Libror AEG-220 analytical balance (Shimadzu Corporation, Japan) was used to prepare standard solutions. Purified deionized water was obtained from Labconco Water Pro-PS UV ultra filtered water system (Labconco Corporation, Missouri). Micro-centrifugation was performed using a BioFuge-Pico D-37520 centrifuge (Heraeus Instruments, Germany).

### Preparation of the calibration standards and the internal standard

Calibration standards were prepared as follows. Gallic acid, caffeine, epicatechin and (−)-epigallocatechin gallate were prepared separately in deionized water to give a concentration of 1 mg/mL. Each solution (5.0 mL) was mixed together in a 100.0 mL volumetric flask and the total volume was adjusted to the mark with deionized water to yield a solution mixture where concentration of each component i.e. gallic acid, caffeine, epicatechin, and (−)-epigallocatechin gallate is equal to 50 μg/mL. This stock solution was diluted accordingly to yield a concentration series of 2.5–25 μg/mL. Stock solution of β-hydroxyethyltheophilline was prepared in deionized water at a concentration of 1 mg/mL. β-hydroxyethyltheophilline (10 μg/mL) was prepared by diluting the stock solution with deionized water and used as the internal standard. The calibration standard mixtures and the internal standard was dispensed separately into sterile centrifuge tubes and stored at −20 °C until further use.

### Preparation of the quality control (QC) materials

Quality control (QC) materials were prepared in deionized water at four concentration levels by spiking known amounts of primary standards of gallic acid, caffeine, epicatechin and (−)-epigallocatechin gallate at concentrations of 3, 4, 7 and 15 μg/mL. QC materials were analyzed for method validation and during sample analysis to ensure the quality of the data. Each QC material was dispensed into 1.5 mL microcentrifuge tubes and stored at −20 °C until further use.

### Determination of GA, Caf, EC and EGCG using reversed phase high pressure liquid chromatography (RP-HPLC)

Chromatographic resolution of components in tea was achieved on a betasil phenyl HPLC column. The mobile phase constituted of isocratic elution system of 8 % acetonitrile, 1 % glacial acetic acid and 91 % deionized water at a flow rate of 0.5 mL/min. The detection wavelength was at 280 nm. β-hydroxyethyltheophilline prepared in deionized water (10 μg/mL) was used as the internal standard. Calibration curves (2.5–25 μg/mL) were constructed with peak area ratio of GA, Caf, EC and EGCG (ratio of peak area of the relevant standard to that of the internal standard) against the concentration for a mixture prepared from the same.

Tea samples were diluted at 1:5 ratio for the quantification of GA, EC and EGCG and 1:50 for the quantification of Caf. Diluted test samples or standards (100 μL) were mixed with β-hydroxyethyltheophilline (10 μg/mL; 100 μL) followed by centrifugation (2000 rpm; 5 min) and the supernatant (25 μL) was injected onto the column. The peaks were identified by comparing the retention times of the components in tea with that of the authentic standards.

### Validation of the RP-HPLC method

The HPLC method was validated according to FDA guide lines (Food and Drug administration 2001). Accuracy, within run and between run precision, linearity, limit of detection (LOD) and limit of quantitation (LOQ) validation parameters were evaluated according to the guidelines provided.

The slope and intercept of the calibration curves were calculated through least squares linear regression analysis using Microsoft Excel Program. The slope and the intercept of the calibration curves were observed over a period of 6 weeks using six independent series of the calibration standards prepared as described earlier.

The calibration plot was accepted if R^2^ > 0.99. QC materials (n = 6) at four concentration levels i.e., 3, 4, 7 and 15 μg/mL prepared similar to the unknown samples were repeatedly measured to determine the accuracy of the measurements. The interday (n = 6) precision of the HPLC method was investigated by repeat analysis of the same QC materials over a period of six successive weeks and evaluating the coefficient of variation (%CV). The within run precision (n = 6) was investigated by repeat analysis of the QC materials at a continuous run within a period 24 h and evaluating the coefficient of variation (%CV). The limit of detection (LOD) and limit of quantification (LOQ) for each of the components measured in tea samples were calculated as follows. Standard error of the y—intercept (δ) and the slope of the calibration curve (S) were determined for the calibration curve of each of the standards. LOD and LOQ were calculated as 3.3 and 10× δ/S respectively (Taylor 1987). Long term stability of the GA, Caf, EC and EGCG was evaluated by repeat analysis of the QC material stored at −20 °C for over a period of 6 weeks. All the experiments were carried out at room temperature.

### Analysis of tea samples

Deionized water (500 mL) was boiled in a glass beaker placed on a magnetic stirrer. At the onset of boiling, heating was stopped and tea leaves (5.0 g) was added to boiled water and beaker was covered with watch glass continuing stirring at a constant speed. Samples (1.0 mL) were withdrawn at 8 min and centrifuged. GA, Caf, EC, EGCG were quantified by the developed reversed phase high pressure liquid chromatography method. Tea brew prepared separately were analyzed in replicates (n = 6).

### Statistical analysis

Results are presented as mean ± standard deviation (Mean ± SD) of six independent experiments. Statistical analysis was performed using Microsoft Excel.
